# History and development of gastroenterology in the Asia Pacific region with particular reference to the role of the Journal of Gastroenterology and Hepatology and the Journal of Gastroenterology and Hepatology Foundation

**DOI:** 10.1002/jgh3.12340

**Published:** 2020-04-26

**Authors:** Khean‐Lee Goh

**Affiliations:** ^1^ Department of Medicine University of Malaya Kuala Lumpur Malaysia

Gastroenterology is the branch of medicine focused on the digestive system and its disorders and broadly includes gastrointestinal (GI) and liver diseases, GI endoscopy, and surgery. The first practitioners of gastroenterology were surgeons. At the turn of the 19th century, surgeons were at the forefront of the treatment of a very common GI disease at that time—peptic ulcer disease.

Gastroenterology developed rapidly as a special field with the advent of fiberoptic endoscopy in the early 1960s. Accurate diagnoses of diseases of the GI tract could then be made with endoscopy. Reflux esophagitis and peptic ulcer disease could be diagnosed with certainty by direct visualization of the pathology. Soon, many endoscopic procedures were performed, and the specialty of gastroenterology started to develop.

In many countries across the world, surgeons started performing endoscopy as part of their clinical practice, but soon, general physicians with a special interest in gastroenterology became more adept and focused at endoscopy than surgeons. As they started to spend more time in gastroenterology and GI endoscopy, the fraternity of gastroenterologists began to be established. Liver diseases were also in their domain. However, with the discovery of the hepatitis viruses and the great advances in the laboratory science of liver diseases, hepatology as a subspecialty started to evolve separately. GI endoscopy had also become more sophisticated, and endoscopists soon had to develop special skills to perform more difficult procedures such as endoscopic retrograde cholangiopancreatography (ERCP) and endoscopic resection of early cancers. GI endoscopy has also now evolved into a separate field.

**Figure 1:**
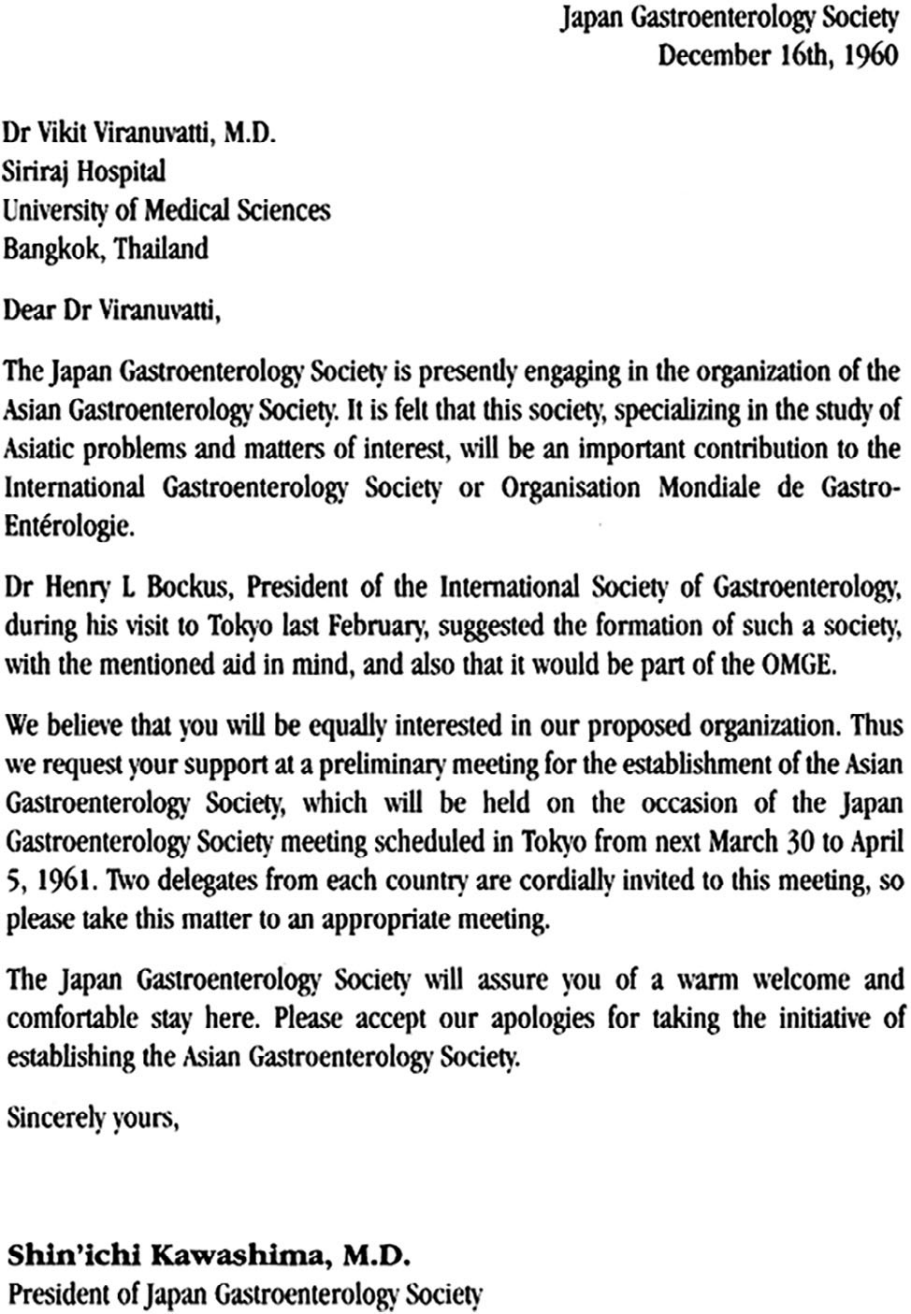
Letter from Dr Shin'ichi Kawashima to Dr Vikrit Viranuvatti

In February 1960, at a meeting in Tokyo, the legendary Henry Bockus of the United States of America, President of the International Society of Gastroenterology or Organisation Mondial de Gastro‐Enterologie (OMGE), had suggested to Dr. Shin‘ichi Kawashima, President of the Japan Society of Gastroenterology, to form an Asian Society of Gastroenterology. The Japan Society had already been in existence for many years! Dr Kawashima gathered his colleagues and friends from across the Asian Pacific region, including Dr Vikrit Viranuvatti of Thailand (Fig. 1), and organized the first congress of the Asian Association of Gastroenterology, as it was originally called, in 1961 in Tokyo. This historical meeting was followed by 4 yearly congresses. In 1972, the name of the group was changed to the Asian Pacific Association of Gastroenterology (APAGE). The Asian Pacific Society of Digestive Endoscopy (A‐PSDE) was formed in 1966 and, in 1972, requested to join the APAGE to hold combined meetings. The joint APAGE/A‐PSDE meetings were successful meetings that attracted large participation from the GI fraternity throughout the region (Table 1). It continued to be held every 4 years.

**Table 1:** The Asian‐Pacific Association of Gastroenterology (APAGE) meetings (from 1972 together with the Asian Pacific Society of Digestive Endoscopy (A‐PSDE)—forerunner of the Asian Pacific Digestive Week (APDW)

	Year	Venue (City)	President of Meeting
Inaugural	1961	Tokyo	Shin'ichi Kawashima
Second	1964	Chandigargh	PN Chuttani
Third	1968	Melbourne	William Morrow
Fourth†	1972	Manila	Pofirio M Recio
Fifth	1976	Singapore	Cheng‐Siang Seah
Sixth	1980	Auckland	Gordon I Nicholson
Seventh	1984	Jakarta	Rudolf Simadibrata
Eighth	1988	Seoul	Chung‐Yong Kim
Ninth	1992	Bangkok	Vikrit Viranuvatti
Tenth	1996	Yokohama	Tadayoshi Takemoto
Eleventh	2000	Hong Kong	Shiu‐Kum Lam

† APSDE with APAGE first Joint Meeting.

The president of the APAGE was the president of the current meeting and held that position until the next meeting 4 years later. It fulfilled the objective of organizing regular scientific meetings, but the association was run in a rather ad‐hoc manner, with few activities in between the meetings. In 2000, Professor Shiu‐Kum Lam from Hong Kong proposed several structural changes to the organization, including that the presidency of the APAGE be delinked from the presidency of the congress. The term of office bearers was also limited to 2 years. This was accepted and passed by the APAGE council in 2000.

The APAGE as an organization, was also officially registered as a legal entity in Hong Kong on 15 June 2001, with a permanent secretariat based in Hong Kong. In the early years, the Hong Kong Society of Gastroenterology allowed the APAGE to utilize part of their office space as the APAGE office.

SK Lam, who was then Dean of Medicine at the University of Hong Kong, proposed an annual Asian‐Pacific Digestive Disease Week (APDW), similar to that of the highly successful Digestive Disease Week of the United States. SK was truly the Asian Pacific leader in gastroenterology at that time. A pro tem committee was set up, including members from sister organizations ‐ the Asian Pacific Study for Liver Disease (APASL) and the A‐PSDE. SK also invited the International Society for Digestive Surgery Asia‐Pacific (ISD‐AP) to join the group, and together, the APDW was born. The group was essentially a “steering committee” with SK Lam as the founding chairman. The APAGE secretariat served as the pro tem secretariat for the group, although the services of a professional congress organizer, “The Meetings Labs” from Singapore, was enlisted to run the “day‐to‐day” work of the committee.

The first APDW was held in 2001 in Sydney under the leadership of Professor Geoff Farrell, who was also at that time the President of the Gastroenterological Society of Australia (GESA). It was a wonderful inaugural meeting held in beautiful new facilities at the Darling Harbour Complex in Sydney. The success, was in no small measure, a reflection of the personal effort and commitment of Geoff Farrell in pushing for a unified Asian Pacific meeting. Subsequently, the meetings were held yearly, rotating between several cities in the region (Table 2). From 2004, KM Fock (Singapore) took over the Chairpersonship of the committee and, with a firm hand, guided the committee through the initial difficult years, when many of the basic guidelines were put in place for the organization of the APDW.

**Table 2:** Asian Pacific Digestive Week (APDW) meetings

	Year	Venue (city)	President of meeting
Inaugural	2001	Sydney	Geoff Farrell
Second	2002	Bangkok	Termchai Chainuvatti
Third	2003	Singapore	Kwong‐Ming Fock
Fourth	2004	Beijing	Shu‐Dong Xiao
Fifth	2005	Seoul	Young‐Il Min, In‐Sung Song
Sixth	2006	Cebu	Jose D Sollano
Seventh	2007	New Delhi	Rakesh Tandon
Eighth	2008	Kobe	Kenji Fujiwara, Hirohumi Niwa
Ninth	2009	Taipei	Cheng‐Shyong Wu
10th	2010	Kuala Lumpur	Khean‐Lee Goh
11th	2011	Singapore	Eng‐Kiong Teo
12th	2012	Bangkok	Udom Kachintorn
13th	2013	Shanghai	Dai‐Ming Fan
14th	2014	Bali	Aziz Rani
15th	2015	Taipei	Jaw‐Town Lin
16th	2016	Kobe	Kentaro Sugano, Michio Kaminishi
17th	2017	Hong Kong	Justin Wu
18th	2018	Seoul	Won‐Ho Kim
19th	2019	Kolkata	Mahesh Goenka

In 2010, a formal constitution was drawn up and accepted by the steering committee, and the APDWF Board was incorporated in Hong Kong as a legal entity. A permanent secretarial office based in Hong Kong was established. KM Fock served as President of the APDWF for two terms until 2014, when Khean‐Lee Goh (Kuala Lumpur) took over as president from 2014 to 2018. Kentaro Sugano (Tochigi) is the current President of the APDWF until 2020. The APDWs have evolved to be very popular and successful meetings. The flexibility given to the local host committee each year to organize the meeting in their respective home cities in the Asian Pacific region has given an interesting diversity and flavor to the meetings while maintaining the high scientific standards that have been set in place by the APDWF.

In 1984, Mark Robertson, Senior Publishing Manager of Blackwell Science based in Australia, first mooted the idea of an Asian Pacific journal dedicated to gastroenterology and hepatology. This provided the impetus to bring together key leaders in the field of gastroenterology. The founding editors of the *Journal of Gastroenterology and Hepatology* (*JGH*) were Shiu‐Kum Lam, Hong Kong; Kunio Okuda, Chiba; Lawrie W Powell, Brisbane; and David JC Shearman, Adelaide, and the first issue was published in 1986.^1^ The young journal was given a big boost when it took abstracts and selected reviews of the World Congresses of Gastroenterology, which was organized by the GESA and held in Sydney from August 26th to 31st 1990. Continuously, from 2001 onwards, abstracts from the annual APDW meetings were also published as supplemental issues of the *JGH*.

**Figure 2:**
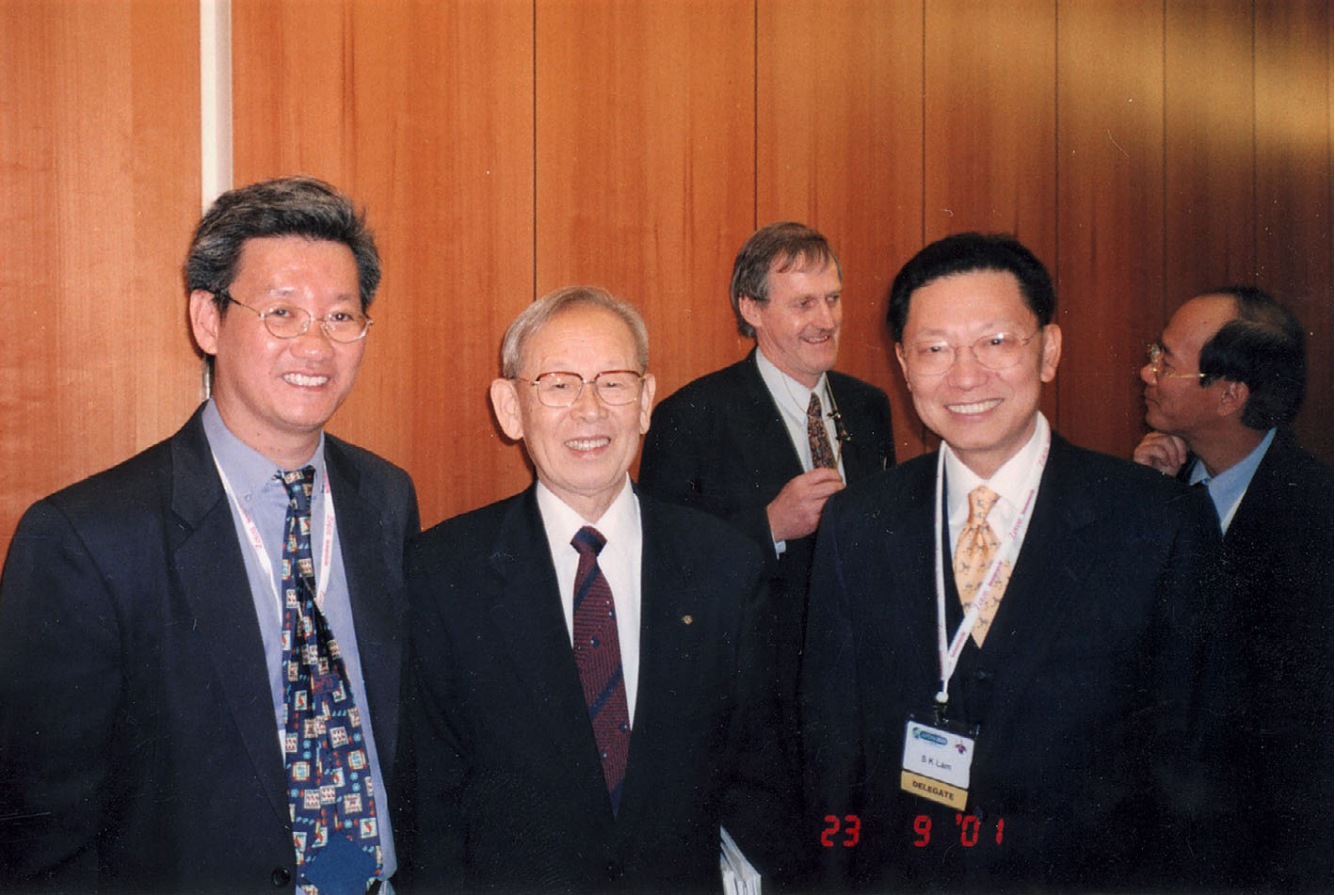
Professors KL Goh, Kunio Okuda, and SK Lam (foreground; left‐right) with professors Ian Roberts Thomson and KM Fock in the background. *JGH* Editor's meeting, 2001.

Kunio Okuda was the inaugural Editor‐in‐Chief, followed by SK Lam (Fig. 2). The journal provided a much‐needed platform for Asian Pacific (AP) contributors to the GI published scientific field in the English language. Although many articles were from the region, the journal soon received top‐class articles from across the world as well.

However, more importantly, the journal brought together a group of senior leaders in the field to cooperate and start meaningful dialogues in collaborative research and in streamlining clinical care across the region. Aside from the principal task of publishing the journal, the editors were, in reality, the leaders of the GI fraternity in the Asian Pacific region and fulfilled an unofficial but important task of weaving together the whole GI community in the region.^2^ The journal has been well run by the publishers, which had changed name over the years: first Blackwell, then Blackwell‐Wiley, and—most recently—Wiley. Mark Robertson remained Head of Publishing in the Asian Pacific region and became a well‐known and respected figure not just within the journal but across the Asian Pacific GI fraternity. His wise counsel through the many years of service in Wiley until his retirement at the end of 2017 has been much appreciated.

**Figure 3:**
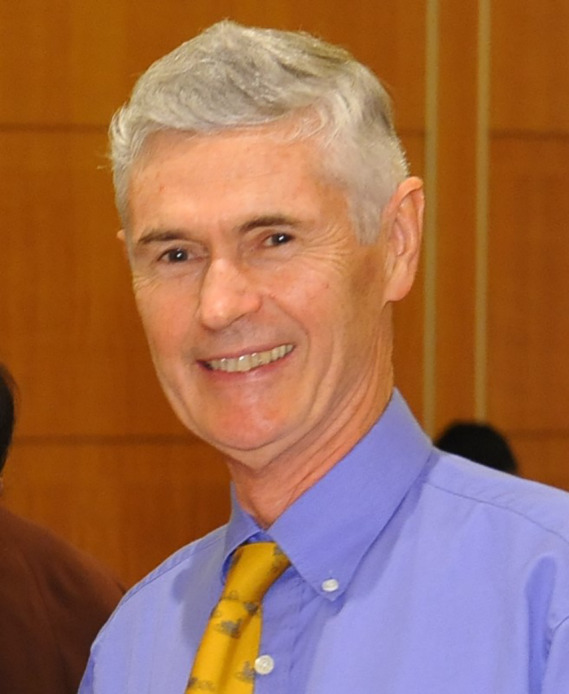
Geoff Farrell, Editor‐in‐Chief, *JGH*, 2006–2011.

Geoff Farrell was editor of the journal from 1991 to 1998. In 2006, he returned to the journal as Editor‐in‐Chief of the *JGH* (Fig. 3), a position that he held until 2011. Through his leadership and his trademark drive and passion, he, more than anyone else, pushed the journal many rungs upward.^3^ He was also instrumental in the establishment of the *JGH* Foundation (originally called the Senior Editors Trust Fund) in 2002. The senior editors at that time decided that the money from the Foundation should be used for the advancement of medicine, medical research and education, and training in the fields of gastroenterology and hepatology within the Asia Pacific region. Its overall aim is to enhance the quality of medical practice and the health of the communities concerned. The trust, the Journal of Gastroenterology and Hepatology Foundation (*JGHF*), was originally set in place as a result of a profit‐sharing agreement between the founding editors and the publisher.^4^ The *JGHF* is registered in Australia as a “company limited by guarantee.” Its nine trustees are drawn from current and past editors of the Journal who serve 3‐year terms that can be renewed for up to a maximum of 9 years. Geoff Farrell was the inaugural chairman from 2002 to 2008. He was followed by Neville Yeoman (Sydney, Melbourne) from 2008 to 2012 and KL Goh (Kuala Lumpur) from 2012 to 2016. From 2016, the Foundation has been in the reliable hands of chairman Ian Roberts Thomson, emeritus professor at University of Adelaide.

The *JGHF* contributes directly to the APDW by fully sponsoring the two main lectures of the meetings, the Warren and Marshall Lecture and the Okuda lecture, as well as two Emerging Leaders lectures. The *JGHF* also collaborates with the APDWF in sponsoring travel grants for young researchers and clinicians to attend the APDW. From 2015, under the leadership of Professor Rakesh Aggarwal (Lucknow), the *JGHF* fully sponsors an exciting 1‐day pre‐congress Young Clinician/Investigator course during the APDW.

In addition, the *JGHF* shares with the APAGE a young Clinician/Scientist Award that was started by Neville Yeomans in 2005 when he was Chairman of the APAGE Awards committee. The *JGHF* also funds numerous projects across the AP region, including symposiums, for example, on celiac disease and FODMAPs and consensus meetings on gastroesophageal reflux diseases, *Helicobacter pylori* infection, and gastric cancer.

The *JGH* Open was inaugurated in 2017 with the first issue being published in September 2017 with KL Goh as the Founding Editor. Initially, it was published monthly, but by 2018, it was switched to a two‐monthly publication schedule. The establishment of the *JGH* Open is in response to the rapidly changing landscape of publishing throughout the world. There are now fewer journals in print but many journals that are published solely online. This saves costs but also expedite publications of articles with free or open access of articles; it is a great boon to researchers and readers. However, the main drawback, at least facing the majority of AP countries, is the article processing charge (APC). Authors and institutions and governments will have to adapt quickly to this change as it is predicted that, by 2025, the majority of major journals across the scientific fields will be published as open access journals. This will be the new norm for journals! For a fledgling journal without an impact factor (IF) and without yet an established profile, this will pose a great challenge in the coming years.

The world of medical publishing has become very competitive. With the retirement of Mamoru Watanabe (Tokyo) in 2017, who had served the *JGH* loyally for 6 years, the *JGH*, in January 2018, appointed a new editor, Joseph Sung from Hong Kong. Joseph, who had recently retired from the position of President and Vice‐Chancellor of the Chinese University of Hong Kong, brings a new spirit of energy and dynamism to the journal with many new and innovative ideas.

The *JGH* and *JGHF* are Asian Pacific icons. Their contribution goes beyond publishing articles. Their far‐reaching influence and respectability, established over the years, will mean that they will continue to play a major role in Asian Pacific gastroenterology for many years to come.
